# Active Surveillance of Adverse Events Following Human Papillomavirus Vaccination: Feasibility Pilot Study Based on the Regional Health Care Information Platform in the City of Ningbo, China

**DOI:** 10.2196/17446

**Published:** 2020-06-01

**Authors:** Zhike Liu, Liang Zhang, Yu Yang, Ruogu Meng, Ting Fang, Ying Dong, Ning Li, Guozhang Xu, Siyan Zhan

**Affiliations:** 1 Department of Epidemiology and Biostatistics Peking University Health Science Center Beijing China; 2 Ningbo Municipal Center for Disease Control and Prevention Ningbo China; 3 National Institute of Health Data Science Peking University Beijing China

**Keywords:** safety, HPV, human papillomavirus, vaccine, active surveillance

## Abstract

**Background:**

Comprehensive safety data for vaccines from post-licensure surveillance, especially active surveillance, could guide administrations and individuals to make reasonable decisions on vaccination. Therefore, we designed a pilot study to assess the capability of a regional health care information platform to actively monitor the safety of a newly licensed vaccine.

**Objective:**

This study aimed to conduct active surveillance of human papillomavirus (HPV) vaccine safety based on this information platform.

**Methods:**

In 2017, one of China’s most mature information platforms with superior data linkage was selected. A structured questionnaire and open-ended interview guidelines were developed to investigate the feasibility of active surveillance following HPV vaccination using the regional health care information platform in Ningbo. The questionnaire was sent to participants via email, and a face-to-face interview was conducted to confirm details or resolve discrepancies.

**Results:**

Five databases that could be considered essential to active surveillance of vaccine safety were integrated into the platform starting in 2015. Except for residents' health records, which had a coverage rate of 87%, the data sources covered more than 95% of the records that were documented in Ningbo. All the data could be inherently linked using the national identity card. There were 19,328 women who received the HPV vaccine, and 37,988 doses were administered in 2017 and 2018. Women aged 30-40 years accounted for the largest proportion. Quadrivalent vaccination accounted for 73.1% of total vaccination, a much higher proportion than that of bivalent vaccination. Of the first doses, 60 (60/19,328, 0.31%) occurred outside Ningbo. There were no missing data for vaccination-relevant variables, such as identity card, vaccine name, vaccination doses, vaccination date, and manufacturer. ICD-10 coding could be used to identify 9,180 cases using a predefined list of the outcomes of interest, and 1.88% of these cases were missing the identity card. During the 90 days following HPV vaccination, 4 incident cases were found through the linked vaccination history and electronic medical records. The combined incident rate of rheumatoid arthritis, optic neuritis, and Henoch-Schonlein purpura was 8.84/100,000 doses of bivalent HPV, and the incidence rate of rheumatoid arthritis was 3.75/100,000 doses of quadrivalent HPV.

**Conclusions:**

This study presents an available approach to initiate an active surveillance system for adverse events following HPV vaccination, based on a regional health care information platform in China. An extended observation period or the inclusion of additional functional sites is warranted to conduct future hypothesis-generating and hypothesis-confirming studies for vaccine safety concerns.

## Introduction

Vaccines, unlike drugs, are generally administered to healthy populations, so the target sample is much larger, especially for birth cohorts, children, or pregnant women. Concerns around vaccine safety, especially for rare adverse events, are increasing with the success of vaccines for the control of vaccine-preventable diseases [1,2]. These concerns can cause a lack of confidence in vaccines and further vaccine hesitancy, defined as a delay in the acceptance or refusal of vaccines despite the availability of vaccination services, that impede or undermine the efforts of an immunization program [3,4]. Although pre-licensure trials can assess the relationship between a vaccine and common adverse events, this type of trial does not have sufficient power to determine the general risk of rare diseases. Therefore, comprehensive safety data from post-licensure surveillance should be used to evaluate the benefit-risk ratio of vaccines, allowing evidence-based decisions by individuals and health organizations [5,6].

Surveillance of adverse events following immunization (AEFI), especially active surveillance for AEFI, should be an essential part of an immunization program to guarantee the safety of vaccines and help establish public confidence [7]. Worldwide, there are a variety of approaches for active AEFI surveillance. Of these approaches, two are the most recognized and replicated due to their robustness and continuity [7]: Vaccine Safety Datalink (VSD), consisting of population-based distributed datasets [8,9], and IMPACT, consisting of targeted hospital-based surveillance [10,11]. These systems were primarily created by developed countries, and limited such systems are available in developing countries [12]. Because of its large population, vast area, and different racial and cultural backgrounds, evidence of vaccine safety in China, especially from active surveillance, can contribute to global vaccine safety efforts.

In recent years, the number of electronic health data sources and interest in their applications have rapidly increased with the advances in medical information systems in China [13], and these electronic health databases could provide the potential for drug safety surveillance and pharmacoepidemiology [14]. Among the electronic health data sources, regional health data sources have been successfully used for drug safety evaluations and surveillance of chronic diseases [15,16]. However, very little is known about the capacity of this data source for active surveillance of adverse events following immunization, and increasing importance has been given to feasibility assessments as essential technical evaluations before starting any vaccine epidemiologic study. Here, we aimed to conduct a feasibility assessment for active surveillance of human papillomavirus (HPV) vaccine safety using the regional health data platform in the city of Ningbo, including a description of the database stakeholders and components, evaluation of the completeness of data relevant to HPV vaccine safety, and an examination of the linkage of data between different data sources.

## Methods

### Study Setting and Data Sources

Ningbo is a city in the Zhejiang province of China (28°51’ – 30°33’ N, 120°55’ – 122°16’ E). It is located in the south wing of the Yangtze River Delta and consists of 4 counties (Yuyao, Cixi, Ninghai, and Xiangshan) and 6 districts (Yinzhou, Haishu, Jiangbei, Zhenhai, Beilun, and Fenghua), with a total land area of 9365 km^2^. In 2017, the city population was >8 million people, and nearly 6 million are part of the hukou (ie, registration of an individual) of Ningbo. The resident population is relatively stable, with a 4.1% out-migration rate and 11.1% in-migration rate.

The regional health care information platform in Ningbo was initially devised and launched in 2011 by the Health Commission of Ningbo to construct a united and standardized medical information network and facilitate health care services. By 2015, the database covered almost all health-related activities of residents within this area, and more than 87% of the residents were registered in the database with a valid health care identifier. In 2016, the database was approved and awarded a Four Grade Class A, which at the time was the top achievement level by the Standardization and Maturity Measurement of Regional Health Information Interconnection by the National Health Commission of China.

### Questionnaire Design and Field Investigation

Based on information in previous studies, we developed the questionnaire and open-ended interview guidelines to evaluate the feasibility of active surveillance following HPV vaccination using the regional health care information platform in Ningbo. To finalize the questionnaire and guidelines, four experts from pharmacoepidemiology, vaccine safety, medical informatics, and database administration independently reviewed the documents and provided feedback. Then, a 2-step survey was conducted to collect database information from the data owners, management, and other staff. First, we sent the questionnaire, its instructions, a comprehensive description of the study purpose, and the completion deadline to the database staff via email. After receiving the questionnaire responses, we confirmed any potential discrepancies with Ningbo through telephone communication. Second, we conducted face-to-face interviews, using the open-ended interview guidelines, with relevant staff during field visits.

The questionnaire about the database consisted of 7 parts: (1) basic information in the database; (2) information in electronic medical records (EMR) in the database; (3) vaccination registration and information about maternal and child health care in the database; (4) method and possibility of linking data between different data sources; (5) age distribution of HPV vaccine–protected women in 2017; (6) completeness of core variables relevant to active surveillance of vaccine safety in 2017; and (7) crucial outcome information in EMR in 2017.

The interview guideline was mainly composed of 3 parts: (1) general characteristics of the database; (2) general characteristics of the essential information system for active surveillance, including vaccination registration, EMR, maternal and child health care information, cancer registration, death registration; and (3) other several specific questions.

The study was approved by the Peking University Institutional Review Board (IRB 00001052-18016). Moreover, all the tasks were performed by staff of the data owner, and investigators could not access the raw data throughout the study period.

## Results

### Stakeholders and Database Components in Ningbo

The Ningbo database was constructed and is owned by the Ningbo Health Commission (NHC), and the Ningbo Centers for Disease Control and Prevention (CDC) can operate and develop this database with permission from the NHC. There are two primary data sources in the database, including the digital CDC platform and digital hospital platform. The CDC controls the former, which mainly focuses on public health and provides a unified information system for the whole city, including integrated primary health care, chronic or infectious disease surveillance, vaccine registration (children and adults), and death registration. Data in the hospital digital platform are collected during regular clinical services provided in hospitals, such as from EMR, and the information system usually differs by hospital, but the NHC has developed harmonized standards to transform and upload these data, which are in a nearly structured format in the Ningbo database. Moreover, the NHC and CDC have endeavored to improve the data quality in the database through official regulations, upgrading of standards, and regular quality checks. The local CDC has also established an institutional review board to review ethical issues and guarantee privacy protection during the process of scientific research.

### Characteristics of the Data Sources for Active Surveillance of HPV Vaccine Safety

Table 1 summarizes the characteristics of the five data sources related to active surveillance for HPV vaccine safety in the Ningbo database; data from these sources were updated and uploaded into the database daily. First, residents’ electronic health records were created primarily by general physicians when the residents visited medical and health institutions for services; these records currently cover >87% of the permanent residents in Ningbo. Second, all vaccination records, including free vaccines provided by the Expanded Programme on Immunization or vaccines paid for out-of-pocket, for infants and children aged <6 years are registered in the immunization program system. It was a compulsory requirement from the Ningbo CDC beginning in May 2017 that vaccination records for adults be registered. Third, starting in 2015, the platform integrated new and historical EMR data from all public hospitals and most of the large private hospitals in the city. Fourth, the maternal and child health care information system includes data recorded during health care services provided for prenatal examinations, maternal deliveries, and newborn follow-ups (until the infant was 1 year old), which resulted in an increase in the local pregnancy and maternal registration rate to 97% by 2016. Finally, the death surveillance information system primarily includes identity information and medical certificates of the dead and can trace death records outside of the city of Ningbo but inside Zhejiang province.

**Table 1:** Characteristics of the data sources related to HPV vaccine safety surveillance.

Data and population		Year surveillance began		Coverage		Main purpose	
**RHR^a^**							
	Residents	2010		>87% of permanent residents		Basic information, health checks, special population primary care	
**IP^b^**							
	Children	2005		100% for 167 vaccination clinics		Basic information, vaccination records, vaccine cold chain	
	Adults	2017					
**EMR^c^**							
	Outpatients^d^	2015		All 221 public health hospitals and 28 large private hospitals		Diagnoses, prescriptions, medical examinations, lab tests	
	Inpatients	2015					
**MCH^e^**							
	Maternal patients	2015		> 97% maternal registration rate		Prenatal examinations, birth deliveries, newborn follow-ups	
	Newborns	2015					
**DS^f^**							
	Residents	2010		Permanent residents		Identity, death certificates	

^a^RHR: residents’ health records.

^b^IP: immunization program.

^c^EMR: electronic medical record.

^d^includes outpatient and emergency visits.

^e^MCH: maternal and child health care.

^f^DS: death surveillance.

### Characteristics of HPV Vaccination in the Immunization Program

From 2017 to 2018, there were two types of HPV vaccines: bivalent and quadrivalent. Both were only available for women in this district. Table 2 describes the characteristics of the population who received the HPV vaccination in Ningbo. During this period, 19,328 women received at least one dose of the HPV vaccine. Women aged 30-40 years represented the largest proportion. Quadrivalent vaccination was administered more often than bivalent vaccination. Moreover, 60 (60/19,328, 0.31%) first doses seemingly occurred outside Ningbo, and these data were not traced back to the database.

The completeness of critical variables relevant to HPV vaccination (eg, recipient identity card [ID], name, gender, birth date, address, vaccine name, vaccination date, vaccination manufacturer, batch number) was also investigated, and for these variables, there were no missing data. The system did not have information for education, occupation, height, weight, smoking habits, and alcohol consumption.

**Table 2:** Characteristics of the women who underwent HPV vaccination.

Variables		Total (n=19,328), n (%)	First dose (n=19,268), n (%)	Second dose (n=13,404), n (%)	Third dose (n=5316), n (%)
**Age group (years)**					
	9-15	1541 (7.97)	1532 (7.95)	1343 (10.02)	613 (11.53)
	16-20	959 (4.96)	943 (4.89)	806 (6.01)	491 (9.24)
	21-25	2735 (14.15)	2721 (14.12)	1961 (14.63)	1064 (20.02)
	26-30	3221 (16.66)	3215 (16.69)	1966 (14.67)	634 (11.93)
	31-35	4146 (21.45)	4138 (21.48)	2554 (19.05)	759 (14.28)
	36-40	4117 (21.30)	4112 (21.34)	2753 (20.54)	902 (16.97)
	41-45	2609 (13.50)	2607 (13.53)	2021 (15.08)	853 (16.05)
**Vaccine**					
	Bivalent	5194 (26.87)	5147 (26.71)	4445 (33.16)	1721 (32.37)
	Quadrivalent	14134 (73.13)	14121 (73.29)	8959 (66.84)	3595 (67.63)

### Identification of the Outcome of Interest in EMR

Table 3 shows the ability of the 10th revision of the International Statistical Classification of Diseases and Related Health Problems (ICD-10) codes to identify the safety outcomes of interest in EMR as well as the incident cases of diseases 90 days after HPV vaccination. All visit records of the cases were retrieved from the EMR using standard query language and the ICD-10 code, and duplicate records were removed. As a result, for data from 2017 and 2018, ICD-10 codes can be used to identify all the outcomes of interest in women aged 9-45 years. Among these diseases, there were 1867 cases of systemic lupus erythematosus, which was the largest proportion of cases. In contrast, there were only 2 cases of acute disseminated encephalomyelitis, which was the smallest proportion of cases. Except for that of Henoch-Schonlein purpura (HSP; 118/1606, 7.35%), the rate of missing IDs for other diseases was near or <5%, and the total rate of missing IDs was 1.88% (173/9180).

Moreover, we monitored the risk of specific adverse events during the 90 days after HPV vaccination. For all the doses, we found 4 incident cases with no history of the disease at least 1 year before HPV vaccination. Two new cases, one each of rheumatoid arthritis (RA) and optic neuritis (ON), were diagnosed within 30 days of bivalent HPV vaccination, and one new case of HSP was diagnosed within 90 days of bivalent HPV vaccination. One new case of RA emerged within 90 days after quadrivalent HPV vaccination.

Hospitals not included in the database could not diagnose nor treat the outcomes of interest. Regarding case validation, structured data from imaging examinations, laboratory tests, histological examinations, and other medical examinations could be found directly in the database, while nonstructured or free-text information, such as the chief complaint, may have needed to be retrieved from the hospital.

**Table 3:** Outcomes among women aged 9-45 years recorded in electronic medical records.

Disease		ICD-10^a^	n^b^	ID^c^ missing, n (%)	New cases 30 days post-HPV^d^ vaccination, n	New cases 90 days post-HPV vaccination, n
**Rheumatologic/autoimmune diseases**						
	SLE^e^	M32	1867	39 (2.09)	0	0
	RA^f^	M05, M06	2878	72 (2.50)	1	2
	JRA^g^	M08.0	52	3 (5.77)	0	0
**Inflammatory bowel disease (IBD)**						
	Crohn disease	K50	135	6 (4.44)	0	0
	Ulcerative colitis	K51	787	17 (2.16)	0	0
**Autoimmune endocrine conditions**						
	Type 1 diabetes	E10	369	18 (4.88)	0	0
	Autoimmune thyroiditis	E06.3	126	2 (1.59)	0	0
	Graves’ disease	E05.0	362	5 (1.38)	0	0
**Autoimmune neurologic and ophthalmic conditions**						
	Multiple sclerosis	G35	30	0 (0)	0	0
	ADEM^h^	G04.0	2	0 (0)	0	0
	GBS^i^	G61.0	11	0 (0)	0	0
	Neuromyelitis optica	G36.0	35	1 (2.86)	0	0
	ON^j^	H46	346	16 (4.62)	1	1
**Others**						
	ITP^k^	D69.3|4	279	4 (1.43)	0	0
	HSP^l^	D69.0	1606	118 (7.35)	0	1
	Bell’s palsy	G51.0	105	5 (4.76)	0	0
	VTE^m^	I82.8|9	59	3 (5.08)	0	0
	Raynaud’s disease	I73.0	131	1 (0.76)	0	0
Total		N/A^n^	9180	173 (1.88)	2	4

^a^ICD-10: 10th revision of the International Statistical Classification of Diseases and Related Health Problems.

^b^duplicate records were removed, and the number of patients was calculated using an encoded identifier consisting of the identity card or name, gender, birth date, and address.

^c^ID: identity card, which is unique for each individual in China.

^d^HPV: human papillomavirus.

^e^SLE: systemic lupus erythematosus.

^f^RA: rheumatoid arthritis.

^g^JRA: juvenile rheumatoid arthritis.

^h^ADEM: acute disseminated encephalomyelitis.

^i^GBS: Guillain-Barre syndrome.

^j^ON: optic neuritis.

^k^ITP: primary immune thrombocytopenia.

^l^HSP: Henoch-Schonlein purpura.

^m^VTE: venous thromboembolism.

^n^N/A: not applicable.

## Discussion

### Principal Findings

A feasibility assessment is the first step to improve the quality of study protocols and accelerate regulatory approvals and, in turn, the start of an actual study [17]. The regional health care information platform in Ningbo covers the entire city and has integrated the essential data sources for active surveillance of the HPV vaccine, including immunization program registration, EMR, resident health records, and death surveillance; these data can be linked using the unique national ID or encoded identifier. More importantly, this database can sensitively identify the outcome of interest through ICD-10 coding, so we can actively monitor risks following HPV vaccination by linking HPV vaccination records with adverse events from EMR, death, or other data sources. Therefore, this database could be available and feasible in research for active surveillance of adverse events following HPV vaccination.

### Comparison With Prior Work

The Chinese CDC established the AEFI Surveillance System, which is a passive national surveillance system covering more than 29 provinces, in 2015 [18]. However, regarding active surveillance, the relevant studies have used traditional methods for timely collection of adverse events, such as by telephone or daily cards, that were designed and performed during the H1N1 pandemic [19]. Plus, we have not found a study that attempted to construct a sustained active surveillance system for AEFI despite the large population and vast geographical area in China [7]. Internationally, some countries have developed sustainable active surveillance systems for adverse events using population-based databases that link vaccine history with outcomes, such as the VSD [9] and Post-Licensure Rapid Immunization Safety Monitoring program [20]. In this study, considering that the crucial data components in the Ningbo database are similar to those in the VSD and Post-Licensure Rapid Immunization Safety Monitoring program, we consider this to be pioneering work in China – to construct a continuous method for actively monitoring AEFI during the post-marketing phase of vaccines. Moreover, we can also link vaccination history with death records in the Ningbo database, which might reduce potential bias from right-censored data.

In this study, of the specific list of outcomes, we identified 4 incident cases within the 90 days following HPV vaccination. The combined incident rate of RA, ON, and HSP was 8.84/100,000 doses of bivalent HPV, and the incident rate of RA was 3.75/100,000 doses of quadrivalent HPV. There was no cluster of these adverse events. Considering the prevalence of RA is 0.46% [21] and the lack of large epidemiology studies of ON [22] and HSP in Chinese women, the rate of adverse events was not high enough for a safety signal. Furthermore, an updated systematic review concluded that the risk of RA, ON, or HSP is not increased with any type of HPV vaccine [23]. However, due to the limited sample size and different backgrounds of Chinese women, a larger sample or longer observational period is required to comprehensively review the safety of HPV vaccines in China.

To monitor the long-term safety of HPV vaccines, a continuous, sustainable surveillance system is necessary. There are several ways of setting controls to promptly identify and alert of vaccine safety signals. First, comparing with historical controls, or background rates, is an approach to identify whether the incidence rate of adverse events following vaccination is higher than the background rate, which is not due to chance [24]. However, it may not be feasible to perform this comparison in the Ningbo database due to the short premarketing period of the HPV vaccine. Four study designs were recently recognized and have become widespread for vaccine safety surveillance, including the self-controlled case series (SCCS), self-controlled risk interval (SCRI), cohort, and case-control study designs [25], and SCRI and SCCS have been proven to be an efficient, rational alternative to the cohort in a simulation study [25,26]. In a real-world study, self-controlled analyses, such as those used in SCRI and SCCS, are used because each case acts as its own control within a short period, thereby inherently adjusting for all time-unvarying potential confounders, such as sex, nationality, and genetic predisposition [27,28]. In our study, given that the database can only capture the visit records of safety outcomes and HPV vaccines that occurred in the city, it is likely that we will misclassify these crucial variables in a subsequent study; for example, some local young women can be vaccinated while attending college outside the city. In addition, these records cannot currently be traced back to the Ningbo database unless the women return to Ningbo and utilize the relevant local services.

Therefore, using the SCRI as the primary design to evaluate the risk after vaccination seems most appropriate, minimizing misclassification bias due to incomplete vaccine exposure. A cohort study as the secondary design may improve statistical power [28]. Also, additional regional databases need to be incorporated to increase the robustness of risk estimation, especially for different subpopulations or districts.

### Limitations

Some potential barriers need to be overcome in additional studies. First, because the HPV vaccination rate is so low in Ningbo, the observation period should be lengthened, or additional sites should be incorporated. Second, identification algorithms for safety outcomes should be developed to improve performance, with a positive predictive value >70% [29]. Third, migration may cause potential bias in the representative population or misclassification of exposure and outcomes, so we should consider its influence in future research. Finally, missing links between data should be addressed and balanced with privacy concerns [30].

### Conclusions

The study presents an available approach to initiate an active surveillance system for adverse events after HPV vaccination, based on a regional health care information platform in China. Utilizing a longer observation period or including additional functional sites is warranted to conduct future hypothesis-generating and hypothesis-confirming studies to address vaccine safety concerns.

## Abbreviations

ADEM: acute disseminated encephalomyelitis.
AEFI: adverse events following immunization.
CDC: Centers for Disease Control and Prevention.
DS: death surveillance.
EMR: electronic medical record.
GBS: Guillain-Barre syndrome.
HPV: human papillomavirus.
HSP: Henoch-Schonlein purpura.
ICD-10: 10th revision of the International Statistical Classification of Diseases and Related Health Problems.
ID: identity card.
IP: immunization program.
ITP: primary immune thrombocytopenia.
JRA: juvenile rheumatoid arthritis.
MCH: maternal and child health care.
N/A: not available.
NHC: Ningbo Health Commission.
ON: optic neuritis.
RA: rheumatoid arthritis.
RHR: residents’ health records.
SCCS: self-controlled case series.
SCRI: self-controlled risk interval.
SLE: systemic lupus erythematosus.
VSD: Vaccine Safety Datalink.
VTE: venous thromboembolism.
